# The Efficacy of Mobile Phone Apps for Lifestyle Modification in Diabetes: Systematic Review and Meta-Analysis

**DOI:** 10.2196/12297

**Published:** 2019-01-15

**Authors:** Xinghan Wu, Xitong Guo, Zhiwei Zhang

**Affiliations:** 1 eHealth Research Institute School of Management Harbin Institute of Technology Harbin China; 2 Department of Statistics University of California at Riverside Riverside, CA United States

**Keywords:** smartphone, mobile applications, diabetes mellitus, lifestyle, physical activity, diet, behavior therapy

## Abstract

**Background:**

Diabetes and related complications are estimated to cost US $727 billion worldwide annually. Type 1 diabetes, type 2 diabetes, and gestational diabetes are three subtypes of diabetes that share the same behavioral risk factors. Efforts in lifestyle modification, such as daily physical activity and healthy diets, can reduce the risk of prediabetes, improve the health levels of people with diabetes, and prevent complications. Lifestyle modification is commonly performed in a face-to-face interaction, which can prove costly. Mobile phone apps provide a more accessible platform for lifestyle modification in diabetes.

**Objective:**

This review aimed to summarize and synthesize the clinical evidence of the efficacy of mobile phone apps for lifestyle modification in different subtypes of diabetes.

**Methods:**

In June 2018, we conducted a literature search in 5 databases (Cochrane Central Register of Controlled Trials, MEDLINE, Embase, CINAHL, and PsycINFO). We evaluated the studies that passed screening using The Cochrane Collaboration’s risk of bias tool. We conducted a meta-analysis for each subtype on the mean difference (between intervention and control groups) at the posttreatment glycated hemoglobin (HbA_1c_) level. Where possible, we analyzed subgroups for short-term (3-6 months) and long-term (9-12 months) studies. Heterogeneity was assessed using the I^2^ statistic.

**Results:**

We identified total of 2669 articles through database searching. After the screening, we included 26 articles (23 studies) in the systematic review, of which 18 studies (5 type 1 diabetes, 11 type 2 diabetes, and 2 prediabetes studies) were eligible for meta-analysis. For type 1 diabetes, the overall effect on HbA_1c_ was statistically insignificant (*P*=.46) with acceptable heterogeneity (I^2^=39%) in the short-term subgroup (4 studies) and significant heterogeneity between the short-term and long-term subgroups (I^2^=64%). Regarding type 2 diabetes, the overall effect on HbA_1c_ was statistically significant (*P*<.01) in both subgroups, and when the 2 subgroups were combined, there was virtually no heterogeneity within and between the subgroups (I^2^ range 0%-2%). The effect remained statistically significant (*P*<.01) after adjusting for publication bias using the trim and fill method. For the prediabetes condition, the overall effect on HbA_1c_ was statistically insignificant (*P*=.67) with a large heterogeneity (I^2^=65%) between the 2 studies.

**Conclusions:**

There is strong evidence for the efficacy of mobile phone apps for lifestyle modification in type 2 diabetes. The evidence is inconclusive for the other diabetes subtypes.

## Introduction

### Background

Diabetes mellitus is a chronic disease that has a negative effect on people’s quality of life and results in a series of unfavorable outcomes [1]. Diabetes mellitus can be divided into three subtypes (type 1 diabetes mellitus [T1DM], type 2 diabetes mellitus [T2DM], and gestational diabetes mellitus [GDM]), which share the same behavioral risk factors, such as inactivity and unhealthy diets [2]. A guideline released by the International Diabetes Federation in 2017 estimated a yearly cost of US $727 billion globally due to diabetes and related complications [2,3]. Efforts in lifestyle modifications, such as daily physical activity and healthy diets, can reduce the risk of prediabetes, improve the health level of people with diabetes, and prevent complications [4-6]. Lifestyle modification is commonly performed through face-to-face consultations at medical institutions, periodic monitoring by rehabilitation specialists, or both. Other, and more personalized, types of lifestyle modifications, such as personal coaching for physical activity interventions and personal consultations for healthy diet interventions, are not widely available [4].

Mobile phone apps are widely used in both developed and developing countries and have shown great potential to deliver personalized medical advice. In prior studies, apps were demonstrated to facilitate patients’ health promotion by improving their self-management awareness and compliance [7-9]. In practice, apps have been used to help people living with various health conditions and problems, including mental health [10,11], heart failure [12], and smoking cessation [13,14]. In addition, more than 120 apps are available in iTunes and Google Play for diabetes management [15].

Moreover, apps for diabetes management have shown great promise toward improving mental and physical health. Research has shown that the use of apps has statistically significant effects in improving self-efficacy, increasing disease knowledge, enhancing physician-patient communication, and lowering diabetes incidence through delivering information, education, self-management, therapeutic advice, and drug guidance [16].

### Objective

Despite growing interest in the efficacy of apps for lifestyle modification in diabetes management, it is unclear what evidence is available and what this evidence suggests. This lack of knowledge hampers the development of practical guidelines on the use of apps for lifestyle modification in the specific types of diabetes. This review aimed to summarize and synthesize the clinical evidence about the efficacy of mobile phone apps for lifestyle modification in the different subtypes of diabetes.

## Methods

### Data Sources and Search Strategy

We conducted a systematic review and report the results according to the guidance of the Preferred Reporting Items for Systematic Reviews and Meta-Analyses (PRISMA) [17]. To identify relevant studies, we systematically searched 5 bibliographic databases: the Cochrane Central Register of Controlled Trials in the Cochrane Library, MEDLINE (via the Web of Science), Embase, CINAHL (via EBSCOhost), and PsycINFO (via EBSCOhost). Multimedia Appendix 1 presents the search strategy for each database based on Boolean operators. The scope of the search was defined by publication dates between January 1, 2006 and May 14, 2018 with no restrictions on the languages used.

### Inclusion and Exclusion Criteria

We included studies if they met all of the following conditions: (1) participants had T1DM, T2DM, or GDM or were prediabetes patients; (2) participants were 18 years of age or older; (3) the study included interventions that used apps as a major component; (4) lifestyle modification (eg, physical activity and healthy diets) was provided via apps; (5) the study measured the participants’ glycated hemoglobin (HbA_1c_), weight, fasting blood sugar, body mass index, or other health-related outcomes; (6) the study was a randomized controlled trial; and (7) the full text of article was available. If 1 or more of the inclusion criteria were not met, we excluded such studies.

Reviewers (XW and XG) searched the extant literature and assessed the studies independently. Any disputes were discussed with a third reviewer (ZZ) to reach a consensus.

### Study Selection

We selected studies in 3 phases: identification, preliminary screening, and full-text screening. In the identification phase, the first author conducted electronic searches. The titles and abstracts of all articles identified were collated into 1 database. In the preliminary screening phase, duplicated records were removed. Two authors (XW and XG) independently screened the titles and abstracts of the identified articles according to the inclusion criteria. Studies that did not meet all inclusion criteria were excluded. When disputes arose, a third author (ZZ) was asked to arbitrate. Relevant reviews were retained in the preliminary screening phase. In the full-text screening phase, the same 2 authors (XW and XG) independently screened the full text in accordance with the stated inclusion criteria. Thereafter, XW and XG hand searched the reference lists of all relevant studies for additional relevant ones. In cases of disagreement, ZZ participated to achieve consensus.

### Data Extraction

XW and XG independently extracted data from the acceptable studies (which had passed the full-text screening) and incorporated them into a spreadsheet. The data extracted included details of the studies such as author, year, country, sample size, study design, diabetes subtype, details of intervention and control, outcomes of interest, and the key results. As in previous reviews and meta-analyses, if a study had multiple intervention arms, we limited data extraction to the most active intervention arm based on the use of apps (ie, the intervention arm that provided the largest collection of interventions based on apps). If the desired data had not been reported in an article, we contacted the first author of the article to retrieve any missing information. In some cases, we back-calculated unreported standard deviations from reported data such as confidence intervals [18].

### Quality Assessment

Two reviewers (XW and XG) independently assessed the risk of bias in the eligible studies in accordance with The Cochrane Collaboration’s risk of bias tool [19]. The instrument has been widely used in evidence-based medical research to evaluate the risk of bias in 6 different aspects (selection, performance, detection, attrition, reporting, and others). In the case of a dispute, we invited another one of the authors to participate in the discussion to help resolve this dispute. we then exported the results of the risk of bias assessment to the software Review Manager (RevMan) version 5.3 (The Nordic Cochrane Centre, The Cochrane Collaboration) to create a visual representation for publication. We handled difficulties in scoring some of the studies by reading the protocols if available (either published in journals or at clinicaltrials.gov, or obtained directly from the authors of each study).

### Data Synthesis and Statistical Analysis

The first author extracted all the data from the appropriate studies. All authors evaluated the preliminary results of the reviews. We conducted a meta-analysis on the mean posttreatment HbA_1c_ values for the intervention and control groups with standard deviations. For studies that only reported mean changes from the baseline, we used this in addition to standard deviations for both groups. If adjusted and unadjusted estimates of treatment effects were both presented, we chose to use the adjusted estimate as reported in the article. When an intention-to-treat and per-protocol analysis were both presented, we chose to use the intention-to-treat results for better internal validity.

We assessed heterogeneity using the I^2^ statistic. We used a fixed effects model when I^2^ was less than 40%; otherwise, we used a random effects model. We conducted subgroup analyses on the long-term (9-12 months) and short-term (3-6 months) effects for each type of diabetes (to the extent possible). All analyses were performed in R version 3.3.3 (R Foundation).

### Outcome Measures

The primary outcome of interest was HbA_1c_. Secondary outcomes included body mass index, weight loss, change in waist circumference, and behavioral changes in physical activity and healthy diets. Physical activity could be measured by step counts, walking activity, and gait performance. Healthy diets were measured by diet balance, food intake, nutrition consumption, and changes in intestinal microflora. Both physical activity and healthy diets were measured using standardized questionnaires.

## Results

### Identified and Included Studies

**Figure 1:**
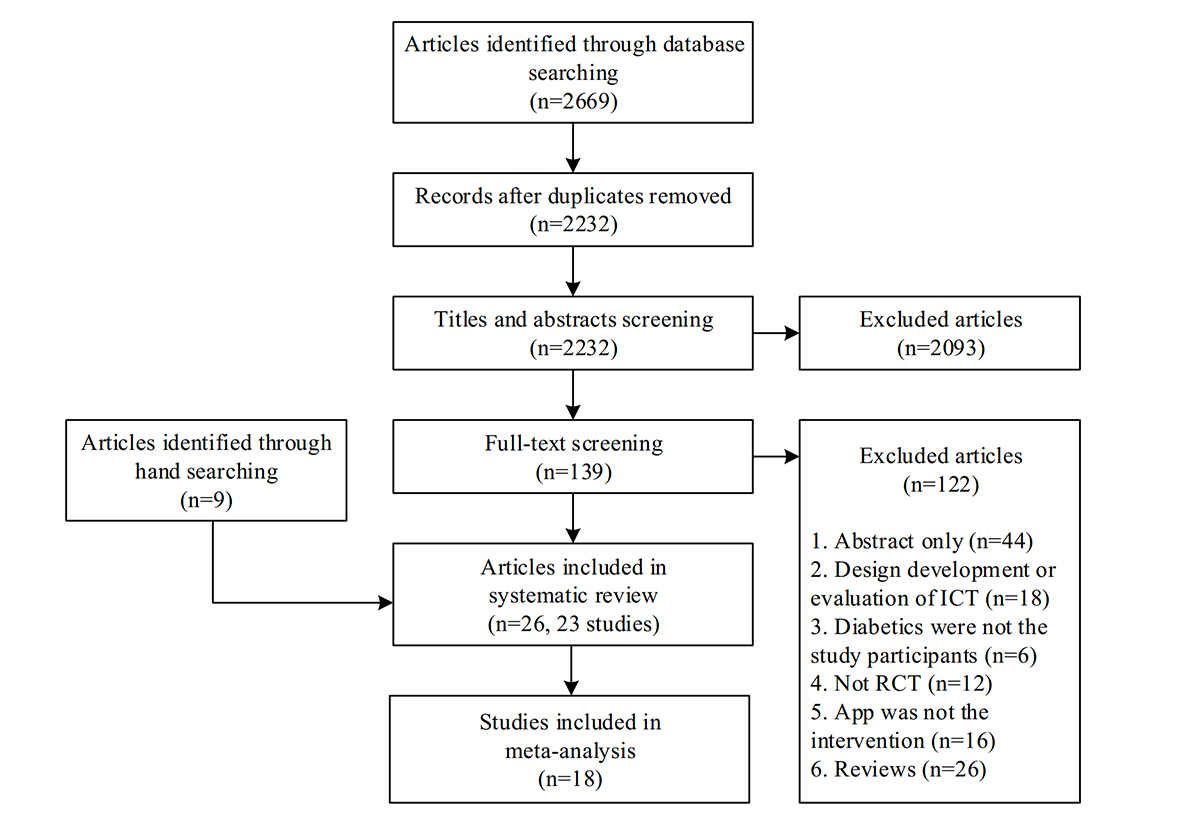
Preferred Reporting Items for Systematic Reviews and Meta-Analyses flowchart of included studies. ICT: information and communication technology; RCT: randomized controlled trial.

The PRISMA diagram in Figure 1 shows our search process and results. We identified a total of 2669 articles through database searching. After we eliminated duplicates, 2232 articles were left for the preliminary screening. In the preliminary screening, we excluded 2093 articles for not meeting all inclusion criteria, leaving 139 articles for full-text screening. Following the full-text screening, we included 17 articles in this review. Separately, we included 9 articles through hand-searched reference lists. Finally, we included 26 articles (based on 23 different studies) in the systematic review, and 18 studies were eligible for meta-analysis. Of the 18 studies eligible for meta-analysis, 5 [20-24] examined T1DM, 11 [25-35] examined T2DM, and 2 [36,37] examined prediabetes. We excluded 1 study [38], which included both T1DM and T2DM patients, from the meta-analysis because the article did not stratify participants according to disease type in reporting efficacy data.

### Study Characteristics

Multimedia Appendix 2 shows the characteristics of all 23 studies. All the studies were randomized controlled trials with apps as the main component of the intervention. In our review, a total of 2526 participants were enrolled. (We included only 1 intervention arm when a study had multiple intervention arms.) In the T1DM studies [20-24], the mean age of participants ranged from 34.9 (SD 13.1) to 39.7 (SD 10.8) years. Of these studies, 4 were undertaken in Europe [20-23] and 1 in Australia [24]. In the T2DM studies [25-35,39,40], the mean age of participants was much higher, ranging from 44.7 (SD 14.0) to 66.3 (SD 8.6) years. Of these studies, 5 were undertaken in Europe [26-28,32,39], 4 in North America [25,29,30,33], 3 in Asia [31,34,35], and 1 in Australia [40]. The 3 prediabetic studies [36,37,41] were undertaken in the United States, and the mean ages of the participants were 40.3 (SD 10.8), 55.2 (SD 9.0), and 55.0 (SD 8.9) years, respectively. Only 1 study focused on pregnant women at high risk of GDM [42], and this study was conducted in the United States, with participants having a mean age of 32.4 (SD 4.4) years. One study [38] enrolled both T1DM and T2DM patients in China with a mean age of 54.3 (SD 12.7) years [38]. The 23 studies ranged from 3 months to 1 year in terms of participant follow-up and investigated the efficacy of apps with respect to physical activity, healthy diets, physiological measures, physical measures, and quality of life. A total of 19 studies measured HbA_1c_ outcomes at the baseline, at posttreatment, or both time points in both the intervention and control groups; Multimedia Appendix 3 presents the relevant summary statistics. Of the 5 studies [22,25,27,39,41] that had multiple intervention arms based on apps, we included the most active arm in the meta-analysis.

### App Characteristics

Multimedia Appendix 4 identifies and summarizes the apps used in the 23 included studies. We describe these below in greater detail.

#### Type 1 Diabetes

All 5 apps in the T1DM studies had lifestyle modification as a major component and included the self-monitoring of participants’ physical activity and healthy diets. Blood glucose was the only clinical measurement that had to be monitored and uploaded by patients. Of these apps, 4 [20-22,24] required professional input on patient health conditions from health care providers (HCPs) via text messages and telephone calls. The frequency of required HCP feedback ranged from once every week to once every 3 weeks. Other feedback modes, including graphical feedback and automated feedback, were employed to promote holistic awareness and to set personal health goals [20-24]. Because of T1DM’s pathological characteristics, all 5 apps included an insulin bolus calculator or a medication adjustment supporter for glycemic control, or both [20-24]. In addition, 1 study that introduced the use of apps did not place an extra time cost on patients’ self-management processes [22].

#### Type 2 Diabetes

The T2DM studies used 13 apps, 12 of which [25-35,39] were designed to modify patient self-management behavior through at least one type of feedback; 4 of these apps [26,31,33,34] provided HCP feedback when necessary. The clinical measurements logged into the apps were blood glucose [25-35], blood pressure [26,28,31,34], body weight [26,28,30,31,34], and mood [33]. Only 3 of these apps provided adjustment support for medications [25,29,32]. None of these apps had an insulin bolus calculator function. Physical activity monitoring was provided by 10 apps [26-28,30,31,33-35,39,40], and healthy diet monitoring was provided by 6 apps [25,27,29,33-35]. One app integrated context exercises into the physical activity function component and aimed to increase motivation and promote positive physical activity behavior [40]. This app, however, did not support assistance from other personnel or any form of feedback.

#### Gestational Diabetes

Kennelly and colleagues [42] studied an app for pregnant women that provided educational sessions on targeted nutrition and physical activity advice. The research team sent emails every other week to address specific problems. Throughout the study, follow-up hospital visits were carried out to ensure proper delivery of the intervention.

#### Prediabetes

The prediabetes studies used 3 apps [36,37,41] designed to help patients with personal weight management. All 3 apps monitored physical activity and healthy diet behavior. Body weight was the only clinical measurement tracked and recorded by patients themselves. Medication adjustment support and insulin bolus calculation were not specified in the articles. Only 1 app provided HCP feedback on a weekly to monthly basis via personalized messages and phone calls [41].

### Risk of Bias Within Studies

**Figure 2:**
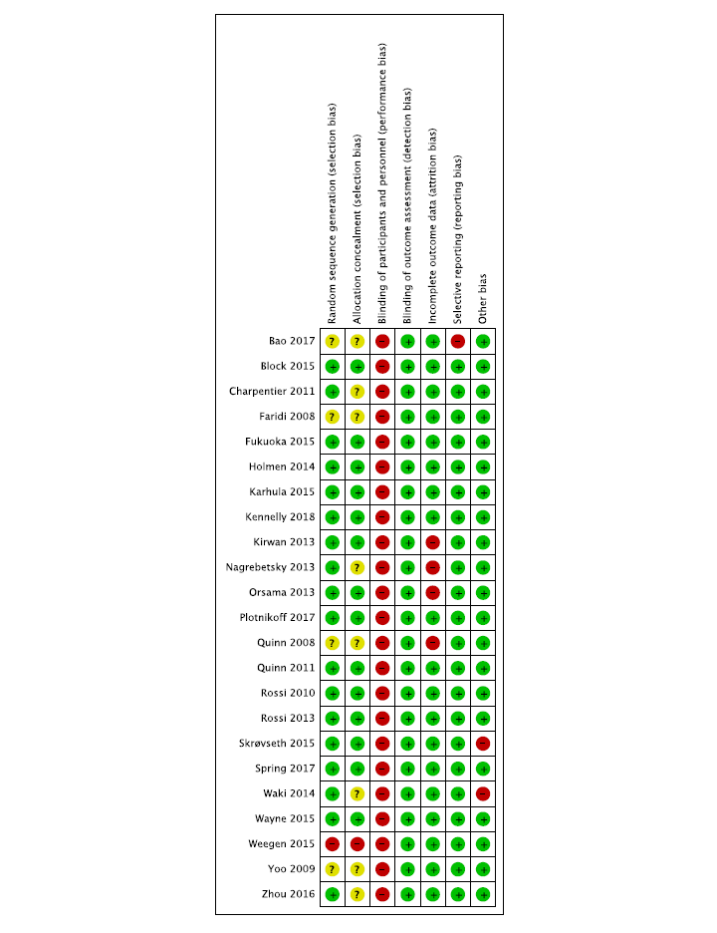
Risk of bias in each study. Green: low risk of bias; yellow: unclear risk of bias; red: high risk of bias.

**Figure 3:**
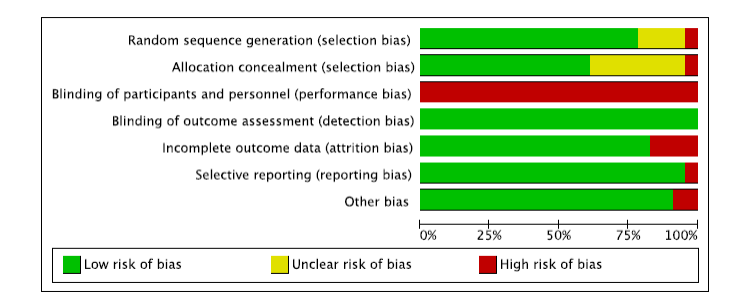
Overall risk of each type of bias.

We assessed the risk of bias in the 23 included studies using The Cochrane Collaboration’s risk of bias tool. In some cases [23-25,27,28,33,36,39-42], we found relevant details in study protocols. Figure 2 (biases by study) and Figure 3 (by overall percentage of each risk) show detailed results. Blinding of participants and personnel is the only area where the risk of bias was high; however, this high risk of performance bias was unlikely to have a large impact on HbA_1c_, whose measurement is fairly objective. In all other areas, the risk of bias was low for most of the studies.

### Efficacy of Apps in Diabetes HbA _1c_ Control

#### Type 1 Diabetes Apps

**Figure 4:**
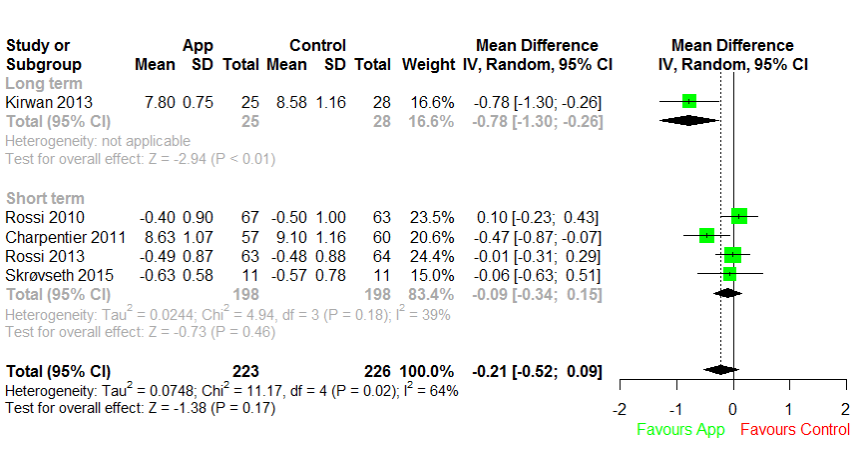
Forest plot of short- and long-term effects of apps for type 1 diabetes mellitus. IV: inverse variance.

We included 5 T1DM studies in the quantitative synthesis, with 4 studies looking at the short-term effect and 1 looking at the long-term effect (Figure 4). In the short-term effect subgroup, the degree of heterogeneity was acceptable (I^2^=39%), and we estimated the overall difference in HbA_1c_ between the app intervention and control groups to be –0.09 (95% CI –0.34 to 0.15), which was not significantly different from 0 (*P*=.18). There was significant heterogeneity in pooling the only long-term effect study with the short-term effect subgroup (I^2^=64%). After pooling, the overall mean difference was statistically insignificant at –0.21 (95% CI –0.52 to 0.09; *P*=.17).

#### Type 2 Diabetes Apps

**Figure 5:**
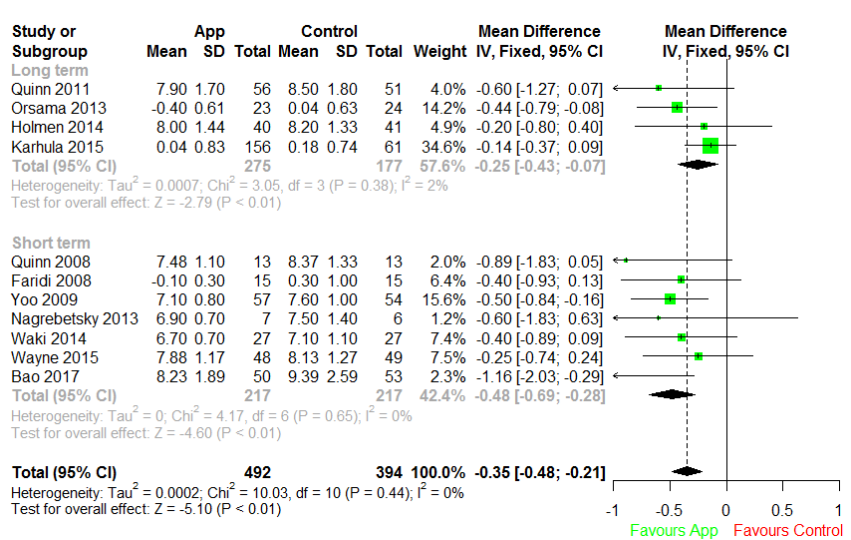
Forest plot of short- and long-term effects of apps for type 2 diabetes mellitus. IV: inverse variance.

We included 11 T2DM studies in the quantitative synthesis, with 7 studies looking at the short-term effect and 4 looking at the long-term effect (Figure 5). In the short-term effect subgroup, there was virtually no heterogeneity (I^2^=0%), and we estimated the overall difference in HbA_1c_ between the app intervention and control groups to be –0.48 (95% CI –0.69 to –0.28), which was significantly different from 0 (*P*<.01). In the long-term effect subgroup, the degree of heterogeneity was acceptable with I^2^=2%, and we estimated the overall difference in HbA_1c_ between the app and control groups to be –0.25 (95% CI –0.43 to –0.07), which was significantly different from 0 (*P*<.01). There was virtually no heterogeneity in pooling the 2 subgroups together (I^2^=0%), and the pooled difference in mean was statistically significant at –0.35 (95% CI –0.48 to –0.21; *P*<.01).

**Figure 6:**
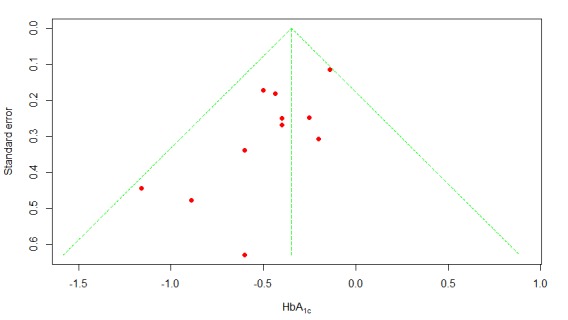
Funnel plot of publication bias. HbA_1c_: glycated hemoglobin.

**Figure 7:**
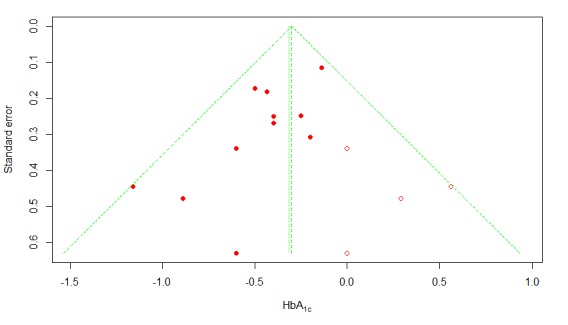
Trim and fill plot of publication bias. HbA_1c_: glycated hemoglobin; open circles: estimated unpublished studies with negative findings.

We used the funnel plot method [43] to examine the publication bias of all 11 T2DM studies. Visual inspection of the funnel plot (Figure 6) revealed a fair amount of asymmetry. Furthermore, Egger and colleagues’ linear regression test [44] was statistically significant (*P*=.02), indicating that the funnel plot was significantly asymmetric, possibly due to publication bias. Therefore, we undertook a sensitivity analysis using the trim and fill method [45] to adjust for possible publication bias in estimating the overall effect size. The trim and fill method estimated that there were 4 unpublished studies with negative findings, shown as open circles in Figure 7. After imputing the 4 unpublished studies, the funnel plot became symmetrical, and the pooled difference in mean HbA_1c_ remained statistically significant at –0.300 (95% CI –0.43 to –0.17; *P*<.001).

**Figure 8:**
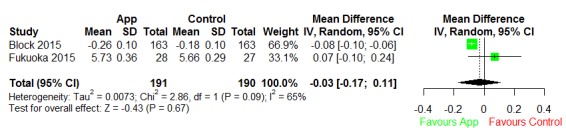
Forest plot the effect of prediabetes apps. IV: inverse variance.

#### Prediabetes Apps

We included 2 prediabetes studies in the quantitative synthesis, and both were short-term studies (Figure 8). Heterogeneity between the studies was significant (I^2^=65%). After pooling, the mean reduction was statistically insignificant at –0.03 (95% CI –0.17 to 0.11; *P*=.67).

## Discussion

### Principal Findings

We included a total of 18 studies with HbA_1c_ outcomes in the quantitative meta-analysis. Among these, there were 5 T1DM studies (4 short-term studies and 1 long-term study). The short-term T1DM studies indicated an insignificant reduction in the HbA_1c_ level with acceptable heterogeneity. The only long-term T1DM study reported a significant reduction in the mean HbA_1c_ level. For T2DM, the meta-analysis included 11 studies, which together showed a significant reduction in the mean HbA_1c_ level, presumably owing to the persuasiveness of the apps for lifestyle modification. In subgroup analyses based on study duration, the long-term and short-term effects were both significant. The long-term effect estimate was slightly smaller than the short-term effect estimate, but the difference was not significant. The meta-analysis also included 2 studies of the prediabetes condition, both of which were of a short-term duration. There was significant heterogeneity between the 2 studies of the prediabetes condition, and the overall difference in the mean HbA_1c_ level between the app and control groups was not statistically significant.

### Comparison With Prior Studies

To the best of our knowledge, this is the first systematic review and meta-analysis with subgroup analyses of apps based on diabetes subtypes and study duration. Previous reviews [46-51] did not include subgroup analyses based on diabetes subtypes and study duration. Some of them [46-48] were limited to one or two specific technologies (eg, pedometer, short message service); others [49,50] addressed different interventions (eg, nonapps and computer-based interventions), and 1 of them [51] focused on usability.

For T1DM, we explored 1 systematic review and meta-analysis published in 2016 [16]. That review found a nonsignificant reduction in the mean HbA_1c_ level of –0.10 (95% CI –0.41 to 0.21) with a high degree of heterogeneity (I^2^=59.15%). Our review included 5 T1DM studies, 4 of which were of short-term duration. Our meta-analysis produced a similar overall effect estimate with less heterogeneity and better precision.

For T2DM, a systematic review and meta-analyses was previously published on the effectiveness of apps for noncommunicable diseases [52]. The 7 studies included in that meta-analysis were T2DM studies of major concern. We also included these studies in our meta-analysis [25-28,33,34]. In the previous reviews, the overall difference in the mean HbA_1c_ level between the app and control groups was estimated to be –0.50 (95% CI –0.91 to –0.08; I^2^=41%) for short-term effects and –0.24 (95% CI –0.43 to –0.06; I^2^=0%) for long-term effects. For the short-term effect, the results are similar to our results in this review. For the long-term effect, our meta-analysis produced a similar effect estimate with less heterogeneity and better precision. An important factor in the decreased heterogeneity and improved precision was the removal of the 1 study that involved both T1DM and T2DM patients [38].

We are not aware of a previous meta-analysis of the overall effect of apps for lifestyle modification in prediabetic patients. However, self-management and continuous care are pivotal issues in prediabetes care for clinical prognosis. Our meta-analysis based on 2 prediabetic studies produced an overall effect estimate of –0.03 (95% CI –0.17 to 0.11). The high degree of heterogeneity between our 2 studies (I^2^=65%) suggests that important effect modifiers may exist for the effects of apps in this context. Searching for effect modifiers should be an important objective for future studies in this area.

As mobile platforms, apps can incorporate different function modules, such as lifestyle modification monitoring, health education, medication or insulin adjustment, logging of clinical measurements, and health management feedback. Feedback, a major behavior change technique, may be implemented as graphical, automated, or HCP feedback. Graphical feedback is probably the most elementary of the three forms; it is frequently used to visualize patient health data [23,24,27,28,30-32,34,36,38,39,41]. Automated feedback is usually provided in a personalized manner. In our review, many studies provided automated feedback based on algorithms or theories [20-22,25-31,34,36-39]. HCP feedback is provided by health care professionals, either in person or remotely [20-22,24-29,31-35,38,39,41,42].

### Limitations

A major limitation of this research was the lack of outcome data beyond 12 months due to the limited duration of the studies reviewed. Diabetes is a chronic condition requiring sustained lifestyle modification, and it is important to understand the longer-term (beyond 12 months) efficacy and safety of apps in our elderly patient population. This gap may be closed by future studies with longer follow-up periods and more complete collection of outcome data (eg, mortality and adverse events). Another limitation is the reliance of some apps on self-reported food intake, which may be unreliable. The effectiveness of such apps in the real world will depend on the quality of user input.

### Conclusion

The results of our review indicate that there is strong evidence for the efficacy of apps for lifestyle modification in T2DM, and that additional evidence is needed for the other subtypes of diabetes. The ambiguous results for T1DM may be related to the pathogenesis of the disease. The efficacy of T1DM self-management is heavily dependent on the administration of glucose with insulin and medication in the short term, which makes it difficult to demonstrate the efficacy of apps. Prediabetes conditions and GDM may be considered transition stages of diabetes, in which the continuum of care can affect the clinical prognosis directly. The different subtypes of diabetes clearly entail different considerations in designing and developing future apps for lifestyle modification in people with diabetes.

## Appendix

Multimedia Appendix 1 Search strategy.

Multimedia Appendix 2 Characteristics of all included studies.

Multimedia Appendix 3 Summary of glycated hemoglobin (HbA_1c_) data by study, treatment arm, and time.

Multimedia Appendix 4 Overview of key functions of the included apps.

## Abbreviations

GDM: gestational diabetes mellitus
HbA_1c_: glycated hemoglobin
HCP: health care provider
PRISMA: Preferred Reporting Items for Systematic Reviews and Meta-Analyses
T1DM: type 1 diabetes mellitus
T2DM: type 2 diabetes mellitus
