# Supplementary material for: The Efficacy of Mobile Phone Apps for Lifestyle Modification in Diabetes: Systematic Review and Meta-Analysis
Source: JMIR Mhealth Uhealth. 2019 Jan 15;7(1):e12297. doi: 10.2196/12297 (PMC6350094; doi:10.2196/12297)
Supplement: Multimedia Appendix 1 [file mhealth_v7i1e12297_app1.pdf]

Multimedia Appendix 1. Search strategy

CENTRAL(159 hits=18/5/14)
#1 (mobile OR mhealth OR "m-health" OR app OR MeSH term Telemedicine OR Cell Phone OR Smartphone OR Mobile Applications)
#2 (diabete* OR T2DM OR TIDM OR IDDM OR NIDDM OR [mh "Diabetes Mellitus"])
#3 ("life style modification" OR activit* OR movement OR motion* OR exercise* OR gait* OR step* OR walk* OR fitness OR diet OR dietary OR eating OR MeSH term Life Style OR Exercise OR Diet, Food, and Nurition)
#4 #1 and #2 and #3

Medline via Web of Science(439hits=18/5/14)
#1 (((((((mobile) OR mhealth) OR "m-health") OR app) OR exp Telemedicine) OR exp Cell Phone) OR exp Smartphone) OR exp Mobile Applications)
#2 (((((diabete*) OR T2DM) OR TIDM) OR IDDM) OR NIDDM) OR exp Diabetes Mellitus)
#3 ((((((((((((("life style modification") OR activit*) OR movement) OR motion*) OR exercise*) OR gait*) OR step*) OR walk*) OR fitness) OR diet) OR dietary) OR eating) OR exp Life Style) OR exp Exercise) OR exp Diet,Food,and Nutrition)
#4 #1 and #2 and #3

Embase(1654 hits=18/5/14)
#1 mobile OR mhealth OR 'm-health' OR app OR 'Cell Phone'/exp OR 'Cell Phone Use'/exp OR 'Mobile Phone'/exp OR 'Smartphone'/exp OR 'Mobile Application'/exp
#2 diabete* OR T2DM OR TIDM OR IDDM OR NIDDM OR 'Diabetes Mellitus'/exp
#3 activit* OR movement OR motion* OR exercise* OR gait* OR step* OR walk* OR fitness OR diet OR dietary OR eating OR 'Diatery Fiber' OR 'Lifestyle Modification'/exp OR 'Physical Activity'/exp OR 'Exercise'/exp OR 'Diet'/exp OR 'Dietary Intake'/exp OR 'Feeding Behavior'/exp OR 'Food'/exp
#4 #1 AND #2 AND #3

CINAHL via EBSCOhost(265hits=18/5/14)
S1 (mobile OR mhealth OR "m-health" OR app OR MH "Telephone+" OR MM "Moble Applications")
S2 (diabete* OR T2DM OR TIDM OR IDDM OR NIDDM OR MH "Diabetes Mellitus")
S3 ("life style modification" OR activit* or movement OR motion* or exercise* or gait* or step* or walk* or fitness OR diet OR dietary OR eating OR MH "Life Style" OR MH "Diet")
S4 S1 and S2 and S3

PsycINFO via EBSCOhost(152hits=18/5/14)
S1 (mobile OR mhealth OR "m-health" OR app OR MH "Telephone+" OR MM

"Moble Applications")
S2 (diabete* OR T2DM OR TIDM OR IDDM OR NIDDM OR MH "Diabetes Mellitus")
S3 ("life style modification" OR activit* or movement OR motion* or exercise* or gait* or step* or walk* or fitness OR diet OR dietary OR eating OR MH "Life Style" OR MH "Diet")
S4 S1 and S2 and S3
