# Supplementary material for: The Efficacy of Mobile Phone Apps for Lifestyle Modification in Diabetes: Systematic Review and Meta-Analysis
Source: JMIR Mhealth Uhealth. 2019 Jan 15;7(1):e12297. doi: 10.2196/12297 (PMC6350094; doi:10.2196/12297)
Supplement: Multimedia Appendix 2 [file mhealth_v7i1e12297_app2.pdf]

Multimedia Appendix 2. Characteristics of all included studies

| First author (year) | Study Design and Intervention Duration | Sample Size | Diabetes Type | Intervention Group and Comparison Group Characterization | Outcomes | Sample Characteristic |
|---|---|---|---|---|---|---|
| Quinn et al (2008) [1] | 2-arm Multicenter RCT; 3 months. | I: N=13 C:N=13 | T2D | IG: 1.Real time feedback on blood glucose levels. 2. Medication regimens display. 3. Hypo- and hyperglycemia treatment algorithms. 4. Access to additional data. CG: NR (Received a glucometer). | $HbA_{1c}$; Change in medication; Diabetes self-care (SDSCA questionnaire). | Age:51.04±11.03 Female:65% Race/Ethnicity:62% AA; 38% white (non-Hispanic) Diabetes Duration: 9.31±NR A1c eligibility:≥7.5% within three month Low Income: NR Setting: Three community physician practices Subject attrition:13.33% |
| Quinn et al (2011) [2, 3] | 4-arm cluster RCT; 12 months. | I:N=56 C:N=51 (12 month | T2D | IG (maximal intervention): 1.Text message reminders and coaching. | $HbA_{1c}$; Diabetes Distress Scale; Diabetes | Age:52.57±8.18 Female:51.28% Race/Ethnicity:37.29 % black (non- |

| First author (year) | Study Design and Intervention Duration | Sample Size | Diabetes Type | Intervention Group and Comparison Group Characterization | Outcomes | Sample Characteristic |
|---|---|---|---|---|---|---|
| | | s) | | 2. Blood and medication monitoring.<br>3. Web portal with secure messaging, medical knowledge database.<br>4. Access to analyzed personal health records<br>CG: Usual care. | symptom inventory;<br>Depression (PHQ-9)<br>Systolic blood pressure;<br>Diastolic blood pressure;<br>LDL;<br>HDL;<br>Triglycerides;<br>Total cholesterol. | Hispanic); 55.08% white (non-Hispanic); 7.63% other<br>Diabetes Duration:8.58±6.15<br>A1c eligibility:≥7.5% within 3 month<br>Low Income: NR<br>Setting: Primary care practice<br>Subject attrition:9.32% (12 month) |
| Kirwan et al (2013) [4] | 2-arm RCT, nine months. | I: N=25<br>C:N=28 | T1D | IG: 1. Personal health records (blood glucose levels, insulin dosages, other medication, diet, physical activity).<br>2. Access to personal data via customizable | $HbA_{1c}$;<br>Summary of Diabetes Self Care Activities (SDSCA, include general diet, specific | Age: 35.20±10.43<br>Female: 61.11%<br>Race/Ethnicity: NR<br>Diabetes Duration: 18.94 ±9.66<br>A1c eligibility:≥7.5%<br>Low Income: NR |

| First author (year) | Study Design and Intervention Duration | Sample Size | Diabetes Type | Intervention Group and Comparison Group Characterization | Outcomes | Sample Characteristic |
|---|---|---|---|---|---|---|
| | | | | graph, and data transfer via email.<br>3. At least one personalized text-message communication per week for the first six months (feedback on logs, diabetes questions, educational tips, positive reinforcement).<br>CG: Usual care (included a visit to primary diabetes health care practitioner every three months) | diet, exercise, glucose testing);<br>Diabetes Empowerment Scale (DES-SF);<br>Diabetes Quality of Life (DQOL);<br>Worry. | Setting: Freely online randomization program<br>Subject attrition: 26.39% |
| Orsama et al (2013) [5] | 2-arm RCT, approximately ten months. | I:N=24<br>C:N=24 | T2D | IG: 1. Personal health-related parameters monitoring (and remote reporting). | Change in $HbA_{1c}$; change in weight; change in | Age:61.90±7.83<br>Female:45.83%<br>Race/Ethnicity: NR<br>Diabetes Duration: |

| First author (year) | Study Design and Intervention Duration | Sample Size | Diabetes Type | Intervention Group and Comparison Group Characterization | Outcomes | Sample Characteristic |
|---|---|---|---|---|---|---|
| | | | | 2. Automatically generated messages (theory based, rich information, behavioral skill feedback) linked to patients' remote reports.<br>3. Access to personal health records.<br>CG: Standard medical care (including diabetes education, annual checkups, diabetes guidance). | systolic blood pressure; change in diastolic blood pressure. | NR<br>A1c eligibility:≥6.5%<br>Low Income: NR<br>Setting: NR<br>Subject attrition: 14.29% |
| Holmen et al (2014) [6-8] | 3-arm RCT, 12 months. | I:N=40<br>C:N=41 | T2D | IG2: 1. Blood glucose level measuring with wireless data transfer;<br>2. Diet manual, physical activity registration, and personal goals management; | $HbA_{1c}$; weight; BMI; Self-management (heiQ); Health-related quality of life (SF-36); | Age:56.73±12.10<br>Female:45.00%<br>Race/Ethnicity: NR<br>Diabetes Duration: NR<br>A1c eligibility:≥7.1%<br>Low Income: NR |

| First author (year) | Study Design and Intervention Duration | Sample Size | Diabetes Type | Intervention Group and Comparison Group Characterization | Outcomes | Sample Characteristic |
|---|---|---|---|---|---|---|
| | | | | 3. Operated using a diabetes diary app;<br>4. Health counseling (based on behavior change theory for the first four months).<br>CG: Usual care (by a general practitioner). | Depressive symptoms (CES-D)<br>Lifestyle changes (dietary habits and physical activity). | Setting: Study centers in collaboration with a general practitioner; and public health clinics in the municipalities.<br>Subject attrition:19.00% |
| Waki et al (2014) [9] | 2-arm RCT, three months | I:N=27<br>C:N=27 | T2D | IG: App (1.Health data monitoring; 2.Exercise and diet information records. 3. Email and lifestyle modification advice; 4.Dietary evaluation).<br>CG: Usual care, continue their self-care regimen. | $HbA_{1c}$;<br>Fasting blood sugar;<br>LDL; HDL;<br>Triglyceride;<br>BMI; Blood pressure;<br>Change in medication;<br>Diabetes self-management (diet, exercise). | Age:57.25±9.72<br>Female:24.07%<br>Race/Ethnicity: NR<br>Diabetes Duration:9.05±7.47<br>A1c eligibility: No limitation<br>Low Income: NR<br>Setting: Home based<br>Subject attrition: 0% |

| First author (year) | Study Design and Intervention Duration | Sample Size | Diabetes Type | Intervention Group and Comparison Group Characterization | Outcomes | Sample Characteristic |
|---|---|---|---|---|---|---|
| Block et al (2015) [10] | 2-arm RCT, 6 months. | I:N=142 C:N=150 | Prediabetes | IG: 1. Tailored behavioral support; 2. Goal setting via emails; 3. Health-related parameters' tracking; 4. Provide relevant information on diabetes; 5. Social support through virtual teams; 6. Modest monetary rewards and team competition; 7. Feedback on goal achievement.<br>CG: Usual-care. | $HbA_{1C}$, change in fasting glucose; weight; BMI; waist circumference; TG/HDL ratio; Framingham diabetes risk score. | Age:55±8.9<br>Female:31.3%<br>Race/Ethnicity: NR<br>Diabetes Duration:67.6% white;6.2% Hispanic;20.6% Asian; 5.6% other<br>A1c eligibility: Fasting glucose or HbA1c in the prediabetes' range<br>Low Income: NR<br>Setting: NR<br>Subject attrition:13.86%(6 months) |
| Fukuoka et al (2015) [11] | 2-arm RCT, 5 months. | I:N=31 C:N=30 | Prediabetes | IG: 1.In-person lifestyle intervention;<br>2. iPhone with the mDPP trial app (short | Weight change; BMI change; $HbA_{1C}$; Other diabetes risk | Age: 55.2±9.0<br>Female:77.0%<br>Race/Ethnicity:22.9% Asian; |

| First author (year) | Study Design and Intervention Duration | Sample Size | Diabetes Type | Intervention Group and Comparison Group Characterization | Outcomes | Sample Characteristic |
|---|---|---|---|---|---|---|
| | | | | educational video clips and texts, daily messages);<br>3.Mobile weight diary and activity/caloric diary, physical activity records, tailored feedback;<br>4. Home-based physical activity plan.<br>CG: Standard medical care. | factors (hip circumference, blood pressure, DBP, lipid panel, glucose), dietary behavior, physical activity, barriers, social support, and depression. | 4.9%African American; 11.5%Hispanic;52.5 %white(non-Hispanic);8.2% more than 1 race<br>Diabetes Duration: NR<br>A1c eligibility:5.7%-7.0%<br>Low Income: NR<br>Setting: Home based.<br>Subject attrition: 0% |
| Karhula et al (2015) [12] | 2-arm RCT, 12 months. | I:N=16 2<br>C:N=63 | T2D and Heart disease | IG: 1.Regular intervals personal health coach (every 4 to 6 months, the coach provided information, assistance, and support to the patients);<br>2.Goal setting based on | Health-related quality of life (SF-36); clinical outcomes (blood pressure, weight, waist circumference, | Age:66.29±8.61<br>Female: 44.4%<br>Race/Ethnicity: NR<br>Diabetes Duration: NR<br>A1c eligibility:≥6.5% within one year<br>Low Income: NR |

| First author (year) | Study Design and Intervention Duration | Sample Size | Diabetes Type | Intervention Group and Comparison Group Characterization | Outcomes | Sample Characteristic |
| --- | --- | --- | --- | --- | --- | --- |
| | | | | patients health condition;<br>3. Personal health recording and health parameters monitoring (data transferred to the individual health records center by SMS automatically or mobile phone once a week);<br>4. Information provided for increasing patients' knowledge of their chronic disease.<br>CG: Standard care (include disease management information booklet, laboratory tests once a year, one appointment or phone call by | triglycerides, total cholesterol, LDL, HDL). | Setting: South Karelia Social and Health Care District (Eksote) in Finland<br>Subject attrition: 10.00% |

| First author (year) | Study Design and Intervention Duration | Sample Size | Diabetes Type | Intervention Group and Comparison Group Characterization | Outcomes | Sample Characteristic |
|---|---|---|---|---|---|---|
| | | | | healthcare givers, freely contact health care services any time). | | |
| Skrøvseth et al (2015) [13] | Randomized stepped-wedge trial with two groups, About five months. (T1:13 weeks, about three months). | I:N=11<br>C:N=11 | T1D | IG:1.Blood glucose data records, daily graph; 2.Data-driven feedback (weekly diagram were presented to the users); 3.Medication reminder; 4.A smartphone application called Diabetes Dairy.<br>CG: A smartphone application called Diabetes Dairy. | $HbA_{1c}$; out of range events. | Age: 39.70±10.8<br>Female:63.33%<br>Race/Ethnicity: NR<br>Diabetes Duration: At least one year<br>A1c eligibility: NR<br>Low Income: NR<br>Setting: NR<br>Subject attrition:27% |
| Wayne et al (2015) [14] | 2-arm RCT, 2 centers, 6 months. | I:N=48<br>C:N=49 | T2D | IG: Health coach of smartphone interaction (1. Decision support: health goals; 2. Action support: goal achievement; 3. | $HbA_{1c}$; weight; waist circumference; BMI; psychometric questionnaires | Age:53.2±11.3<br>Female:72%<br>Race/Ethnicity:1% Frist nations;5% Black: African; 40% Black:Caribbean; |

| First author (year) | Study Design and Intervention Duration | Sample Size | Diabetes Type | Intervention Group and Comparison Group Characterization | Outcomes | Sample Characteristic |
|---|---|---|---|---|---|---|
| | | | | 37min/week (SD 22) of interaction (mean); 4. Samsung Galaxy Ace 2, connected wellness platform account; 5. Tracking/monitoring of meals, exercise, blood glucose, and mood).<br>CG: Health coach without smartphone interaction (1. Decision support: health goals; 2. Action support: goal achievement; 3. 39min/week (SD 28) of interaction (mean); 4. Verbal discussion of meals, exercise, blood glucose, and mood). | (Hospital Anxiety and Depression Scale; Positive and Negative Affect Schedule; Short Form Health Survey-12) | 27% Caucasian; 9% Hispanic; 4% South Asian; 4% South East Asian; 6% West Indian; 3% Other<br>Diabetes Duration: NR<br>A1c eligibility:≥7.3% within one month<br>Low Income: Yes (90% participants were from a lower-income neighborhood, and 10% participants were from a midlevel-SES community)<br>Setting:2 primary health clinics<br>Subject |

| First author (year) | Study Design and Intervention Duration | Sample Size | Diabetes Type | Intervention Group and Comparison Group Characterization | Outcomes | Sample Characteristic |
|---|---|---|---|---|---|---|
| | | | | | | attrition:26% |
| Weegen et al (2015) [15] | 3-arm clustered RCT, 9 months. | I:N=52<br>C:N=65 | T2D and COPD | IG 1: 1. Dialogue sessions and feedback on app and website (Tool);<br>2. Behavioral change consultations in general practice (SSP).<br>CG: Usual care. | Physical activity moderate and vigorous; Self-efficacy (General self-efficacy scale, Exercise self-efficacy scale); Quality of life (RAND physical component, RAND Mental component). | Age:58.37±7.28<br>Female:53.38%<br>Race/Ethnicity:6.02 % Origin non-Dutch<br>Diabetes Duration: NR<br>A1c eligibility: NR<br>Low Income: NR<br>Setting: 24 practices in 2 blocks<br>Subject attrition:12.03% |
| Zhou et al (2016) [16] | 2-arm RCT, 3 months. | I:N=50<br>C:N=50 | T1D and T2D (18 T1D, 82 T2D) | IG: Welltang application (consists of three main parts: knowledge, self-management, and communication between patients and | HbA1c, FBG, 2h-BG, weight, BMI, waist circumference, hip circumference, | Age: 54.25±12.71<br>Female: 43%<br>Race/Ethnicity: NR<br>Diabetes Duration: NR<br>A1c eligibility: NR |

| First author (year) | Study Design and Intervention Duration | Sample Size | Diabetes Type | Intervention Group and Comparison Group Characterization | Outcomes | Sample Characteristic |
|---|---|---|---|---|---|---|
| | | | | clinicians):<br>1. Knowledge of diet, exercise, medicine, blood glucose monitoring and the last guidelines for diabetes care as summaries;<br>2. Self-management, self-care data records, patients have access to their self-care data;<br>3. Communication between patients and clinicians, the study team provided feedback on the blood glucose levels of patients and personalized medication regimens based on patients' data.<br>CG: Usual care (did not | systolic blood pressure, diastolic blood pressure, diabetes knowledge score, self-care behaviors score, hypoglycemic events, LDL-C. | Low Income: NR<br>Setting: Hospital<br>Subject attrition:0% |

| First author (year) | Study Design and Intervention Duration | Sample Size | Diabetes Type | Intervention Group and Comparison Group Characterization | Outcomes | Sample Characteristic |
|---|---|---|---|---|---|---|
| | | | | use the Welltang application). | | |
| Plotnikoff et al (2017) [17] | 2-arm RCT, 5 months. | I:N=28 C:N=32 | T2D (at risk of, or diagnosed with T2D) | IG: 1.Phase 1 (weeks 1-10): (1) face-to-face group sessions which included cognitive mentoring sessions and outdoor PA sessions; (2) eCoFit smartphone app: (a) A description of where and how to use the outdoor physical environment (e.g., park benches) to be physically active; b) 'eCoFit challenges' in 11 different park locations; c) 'Indoor Challenges' which provided aerobic and RT PA sessions that can be completed | Aerobic fitness; lower body muscular fitness, chair stand test; physical activity; functional mobility; upper body muscular fitness, arm curl test; waist circumference; BMI; systolic blood pressure; diastolic blood pressure. | Age: 44.7±14.0 Female:70.2% Race/Ethnicity:83.3% Australian; 3.6% Asian; 8.3% European; 1.2% African; 3.6% Other Diabetes Duration: NR A1c eligibility: including but not limited to HbA1c level >7.0 Low Income: NR Setting: NR Subject attrition:28.57% |

| First author (year) | Study Design and Intervention Duration | Sample Size | Diabetes Type | Intervention Group and Comparison Group Characterization | Outcomes | Sample Characteristic |
|---|---|---|---|---|---|---|
| | | | | indoors or at home; d) A function to set weekly PA goals (options: when, where, with whom, and what I will do to make it happen) and self-monitor the progress; e) 'FitMind Challenges' which involved short tasks to increase motivation, overcome barriers, and develop positive PA behaviors; and f) Links to social media.);<br>2. Phase 2 (week 11-20): eCoFit smartphone app;<br>CG: NR. | | |
| Spring et al (2017) | 3-arm RCT, 12 months. | I:N=31<br>C:N=24 | Obesity | IG 2: 1. Technology-supported treatment | Weight change. | Age: 40.25±10.82<br>Female:85.95% |

| First author (year) | Study Design and Intervention Duration | Sample Size | Diabetes Type | Intervention Group and Comparison Group Characterization | Outcomes | Sample Characteristic |
|---|---|---|---|---|---|---|
| [18] | | | | group (TECH) received eight in-person group treatment sessions; 2. TECH used a smartphone application with social networking features and wireless accelerometer.<br>CG: Self-guided (used paper diaries to self-monitor diet, activity, and weight). | | Race/Ethnicity:14.05 % Hispanic or Latino; 85.95 Other<br>Diabetes Duration: NR<br>A1c eligibility: NR<br>Low Income: NR<br>Setting: NR<br>Subject attrition:14.06% |
| Bao et al (2017) [19] | 2-arm RCT, 3 month. | I:N=50<br>C:N=53 | T2D | IG: 1. Regular dietary intervention as a control group;<br>2. Smartphone application for diabetes management (knowledge about diabetes, daily care, physical activity | FPG; 2h PBG; HbA1c; TC; TG; HDL; LDL. | Age:58.39±11.21<br>Female:46.36%<br>Race/Ethnicity: NR<br>Diabetes Duration: NR<br>A1c eligibility: By Chinese T2D prevention guideline<br>Low Income: NR |

| First author (year) | Study Design and Intervention Duration | Sample Size | Diabetes Type | Intervention Group and Comparison Group Characterization | Outcomes | Sample Characteristic |
|---|---|---|---|---|---|---|
| | | | | records, self-monitoring, personalized diet recommendation based on patients health condition and physicians support).<br>CG: Normal dietary intervention (provided dietary health education handbook). | | Setting: Hospital<br>Subject attrition: 6.36% |
| Kennelly et al (2018) [20] | 2-arm RCT, | I:N=241<br>C:N=257 | Pregnant women (at risk of gestational diabetes mellitus) | IG: The intervention consisted of specific dietary and exercise advice that addressed behavior change supported by a tailor-designed smartphone application.<br>CG: Usual care. | The incidence of GDM (at 28-30 weeks of gestation); gestational weight gain; dietary glycemic index; glycemic load; exercise; mid- | Age:32.44±4.41<br>Female:100%<br>Race/Ethnicity:91.15% Caucasian; 8.85% Other<br>Diabetes Duration: NR<br>A1c eligibility: NR<br>Low Income: NR<br>Setting: Hospital |

| First author (year) | Study Design and Intervention Duration | Sample Size | Diabetes Type | Intervention Group and Comparison Group Characterization | Outcomes | Sample Characteristic |
|---|---|---|---|---|---|---|
| | | | | | upper arm circumference; body fat mass; blood loss at delivery; postpartum hemorrhage; antenatal admissions; maternal lipid profile; glucose homeostasis; prenatal and postnatal characteristics. | Subject attrition:11.86% |
| Rossi et al (2010) [21] | 2-arm RCT, six months | I: N=67<br>C:<br>N=63 | T1D | IG: The intervention (DID system) consists of:<br>1. Blood glucose value records and a dose of insulin injections in real | $HbA_{1c}$; FBG; Systolic blood pressure; Diastolic blood pressure; Total cholesterol; | Age:35.7±9.4<br>Female:56.92%<br>Race/Ethnicity: NR<br>Diabetes Duration: 16.5±10.5<br>A1c eligibility: NR |

| First author (year) | Study Design and Intervention Duration | Sample Size | Diabetes Type | Intervention Group and Comparison Group Characterization | Outcomes | Sample Characteristic |
|---|---|---|---|---|---|---|
| | | | | time;<br>2. The suggestion of daily carbohydrate intake, summing the amount of carbohydrate consumed progressively;<br>3. Short text messages for patients and physicians' communication improvement.<br>CG: Standard care. | HDL cholesterol; LDL cholesterol; Triglycerides; Weight; DTSQ; SF-36 | Low Income: NR<br>Setting: Seven diabetes outpatients clinics<br>Subject attrition:8.46% |
| Rossi et al (2013) [22] | 2-arm RCT, six months | I: N=63<br>C:<br>N=64 | T1D | IG: The intervention consists of:<br>1. Patients had three prandial injections per day of insulin glulisine associated with basal insulin glargine;<br>2. Use DID system, as | $HbA_{1c}$; FBG; Blood glucose; MAGE; Body weight; Insulin mean daily dose; SBP; DBP; Total cholesterol; | Age: 36.9±10.5<br>Female:52,76%<br>Race/Ethnicity: NR<br>Diabetes Duration: 16.3±9.3<br>A1c eligibility: ≥7.5%<br>Low Income: NR<br>Setting: 12 diabetes |

| First author (year) | Study Design and Intervention Duration | Sample Size | Diabetes Type | Intervention Group and Comparison Group Characterization | Outcomes | Sample Characteristic |
|---|---|---|---|---|---|---|
| | | | | previously mentioned.<br>CG: Standard care | HDL-cholesterol; LDL-cholesterol; Triglycerides; DTSQ; DSQOLS | clinics<br>Subject attrition:11.81% |
| Charpentier et al (2011) [23] | 3-arm RCT, six months | I: N=56<br>C: N=60 | T1D | IG: 1. A smartphone loaded with the Diabeo software (record SMPG level before meals, carbohydrate counts, and planned physical activity);<br>2. Face-to-face follow-up visits<br>CG: Standard care (paper logbook, attend two follow-up visits at the hospital) | $HbA_{1c}$; SMPG; insulin dose modification; QOL; | Age:34.87±13.06<br>Female:63.64%<br>Race/Ethnicity: NR<br>Diabetes Duration: 17.25±9.71<br>A1c eligibility: ≥8%<br>Low Income: NR<br>Setting: 17 hospital sites in France<br>Subject attrition:4.13% |
| Faridi et al (2008) | 2-arm RCT, three | I: N=15<br>C: | T2D | IG: The NICHE technology for type 2 | $HbA_{1c}$; BMI; weight; Blood | Age:56±9.55<br>Female:63.33% |

| First author (year) | Study Design and Intervention Duration | Sample Size | Diabetes Type | Intervention Group and Comparison Group Characterization | Outcomes | Sample Characteristic |
|---|---|---|---|---|---|---|
| [24] | months | N=15 | | diabetes management: tailored feedback and reminders based on patient-specific data;<br>CG: Standard diabetes self-management and tracked their step count using a pedometer. | pressure (systolic, diastolic); DSES; SDSC; YPAS; Pedometer step count | Race/Ethnicity: NR<br>Diabetes Duration: NR<br>A1c eligibility: NR<br>Low Income: NR<br>Setting: Primary care network in the USA<br>Subject attrition:0% |
| Nagrebetsky et al (2013) [25] | 2-arm RCT, 12 months | I: N=7<br>C: N-6 | T2D | IG: Intervention group patients followed a stepwise treatment plan for titration of oral glucose-lowering medication with self-monitoring of glycemia using real-time graphical feedback on a mobile telephone and remote nurse monitoring using a Web-based tool; | $HbA_{1c}$ | Age: 58±11<br>Female: 29%<br>Race/Ethnicity:100 % White<br>Diabetes Duration: at least three months<br>A1c eligibility: ≥8.0%, [illegible]11.0%<br>Low Income: NR<br>Setting: general practices in the UK<br>Subject attrition:7.14% |

| First author (year) | Study Design and Intervention Duration | Sample Size | Diabetes Type | Intervention Group and Comparison Group Characterization | Outcomes | Sample Characteristic |
|---|---|---|---|---|---|---|
| Yoo et al (2009) [26] | 2-arm RCT, three months | I: N=57<br>C: N=54 | T2D | CG: Standard care.<br>IG: Intervention group patients use a UCDC system: 1. The UCDC system sent an alarm on the cellular phone to remind the participant to measure their blood glucose, blood pressure, and weight; and automatically sent the results to a central study database. Received messages of encouragement, reminders, and recommendations;<br>2. Automatically recorded participant's exercise time using the SMS messages; | Weight; BMI; Waist circumference; Systolic blood pressure; Diastolic blood pressure; Right baPWV;<br>Left baPWV; $HbA_{1c}$; Fasting glucose; HOMA-IR; Total cholesterol; HDL-cholesterol; LDL-cholesterol; Triglyceride; Adiponectin; hsCRP; | Age: 58.17±8.81<br>Female: 41.44%<br>Race/Ethnicity: NR<br>Diabetes Duration: 6.58±5.71<br>A1c eligibility: 6.5-10.0%<br>Low Income: NR<br>Setting: A university hospital and a community healthcare center<br>Subject attrition:9.76% |

| First author (year) | Study Design and Intervention Duration | Sample Size | Diabetes Type | Intervention Group and Comparison Group Characterization | Outcomes | Sample Characteristic |
|---|---|---|---|---|---|---|
| | | | | 3. Participants received information via SMS three times a day regarding healthy diet and exercise methods, along with general information about diabetes, hypertension, and obesity;<br>CG: Usual care. | Interleukin-6 | |

1. Quinn CC, Clough SS, Minor JM, Lender D, Okafor MC, Gruber-Baldini AL. WellDoc mobile diabetes management randomized controlled trial: change in clinical and behavioral outcomes and patient and physician satisfaction. Diabetes Technol Ther 2008;10(3):160-8. PMID: 18473689.
2. Quinn CC, Shardell MD, Terrin MD, Barr EA, Ballew SH, Gruber-Baldini AL. Cluster-randomized trial of a mobile phone personalized behavioral intervention for blood glucose control. Diabetes care 2011;34(9):1934-42. PMID: 21788632.
3. Quinn CC, Sareh PL, Shardell ML, Terrin ML, Barr EA, Gruber-Baldini AL. Mobile diabetes intervention for glycemic control: impact on physician prescribing. J Diabetes Sci Technol 2014;8(2):362-70. PMID: 24876589.
4. Kirwan M, Vandelanotte C, Fenning A, Duncan MJ. Diabetes self-management smartphone application for adults with type 1 diabetes: randomized controlled trial. J Med Internet Res 2013;15(11):e235. PMID: 24225149.

5. Orsama AL, Lähteenmäki J, Harno K, Kulju M, Wintergerst E, Schachner H, et al. Active assistance technology reduces glycosylated hemoglobin and weight in individuals with type 2 diabetes: results of a theory-based randomized trial. Diabetes Technol Ther 2013;15(8):662-9. PMID: 23844570.
6. Holmen H, Torbjornsen A, Wahl AK, Jenum AK, Smastuen MC, Arsand E, et al. A mobile health intervention for self-management and lifestyle change for persons with type 2 diabetes, part 2: one-year results from the norwegian randomized controlled trial RENEWING HEALTH. JMIR Mhealth Uhealth 2014;2(4):e57. PMID: 25499872.
7. Torbjørnsen A, Jenum AK, Småstuen MC, Arsand E, Holmen H, Wahl AK, et al. A low-intensity mobile health intervention with and without health counseling for persons with type 2 diabetes, part 1: baseline and short-term results from a randomized controlled trial in the Norwegian part of RENEWING HEALTH. JMIR Mhealth Uhealth 2014;2(4):e52. PMID: 25499592.
8. Holmen H, Wahl A, Torbjørnsen A, Jenum AK, Småstuen MC, Ribu L. Stages of change for physical activity and dietary habits in persons with type 2 diabetes included in a mobile health intervention: the Norwegian study in RENEWING HEALTH. BMJ Open Diabetes Res Care 2016;4(1):e000193. PMID: 27239317.
9. Waki K, Fujita H, Uchimura Y, Omae K, Aramaki E, Kato S, et al. DialBetics: a novel smartphone-based self-management support system for type 2 diabetes patients. J Diabetes Sci Technol 2014;8(2):209-15. PMID: 24876569.
10. Block G, Azar K, Romanelli R, Block T, Hopkins D, Carpenter H, et al. Diabetes prevention and weight loss with a fully automated behavioral intervention by email, web, and mobile phone: a randomized controlled trial among persons with prediabetes. J Med Internet Res 2015;17(10):e240. PMID: 26499966.
11. Fukuoka Y, Gay C, Joiner K, Vittinghoff E. A novel diabetes prevention intervention using a mobile app: a randomized controlled trial with overweight adults at risk. Am J Prev Med 2015;49(2):223-37. PMID: 26033349.
12. Karhula T, Vuorinen AL, Rääpysjärvi K, Pakanen M, Itkonen P, Tepponen M, et al. Telemonitoring and mobile phone-based health coaching among finnish diabetic and heart disease patients: randomized controlled trial. J Med Internet Res 2015;17(6):e153. PMID: 26084979.
13. Skrøvseth S, Årsand E, Godtliebsen F, Joakimsen R. Data-driven personalized feedback to patients with type 1 diabetes: a randomized trial. Diabetes Technol Ther 2015;17(7):482-9. PMID: 25751133.
14. Wayne N, Perez D, Kaplan D, Ritvo P. Health coaching reduces HbA1c in type 2 diabetic patients from a lower-socioeconomic status community: a randomized controlled trial. J Med Internet Res 2015;17(10):e224. PMID: 26441467.

15. Weegen S, Verwey R, Spreeuwenberg M, Tange H, Weijden T, Witte L. It's LiFe! Mobile and web-based monitoring and feedback tool embedded in primary care increases physical activity: a cluster randomized controlled trial. J Med Internet Res 2015;17(7):e184. PMID: 26209025.
16. Zhou W, Chen M, Yuan J, Sun Y. Welltang – a smart phone-based diabetes management application – improves blood glucose control in Chinese people with diabetes. Diabetes Res Clin Pract 2016;116:105-10. PMID: 27321324.
17. Plotnikoff RC, Wilczynska M, Cohen KE, Smith JJ, Lubans DR. Integrating smartphone technology, social support and the outdoor physical environment to improve fitness among adults at risk of, or diagnosed with, type 2 Diabetes: findings from the 'eCoFit' randomized controlled trial. Preventive Medicine 2017;105:404-11. PMID: 28887192.
18. Spring B, Pellegrini CA, Pfammatter A, Duncan JM, Pictor A, McFadden HG, et al. Effects of an abbreviated obesity intervention supported by mobile technology: the ENGAGED randomized clinical trial. Obesity 2017;25(7):1191-8. PMID: 28494136.
19. Bao S, Jiang H, Luo Y, Zhang D. Application of diabetes phone recipe software in diet intervention for patients with type 2 diabetes. Chinese Nursing Research 2017;31(11):1407-8. Doi: 10.3969/j.issn.1009-6493.2017.11.042.
20. Kennelly MA, Ainscough K, Lindsay KL, O'Sullivan E, Gibney ER, McCarthy M, et al. Pregnancy exercise and nutrition with smartphone application support: a randomized controlled trial. Obstet Gynecol 2018;131(5):818-26. PMID: 29630009.
21. Rossi MCE, Nicolucci A, Bartolo PD, Bruttomesso D, Girelli A, Ampudia FJ, et al. Diabetes interactive diary: a new telemedicine system enabling flexible diet and insulin therapy while improving quality of life: an open-label, international, multicenter, randomized study. Diabetes Care 2010;33(1):109-15. PMID: 19808926.
22. Rossi MCE, Nicolucci A, Lucisano G, Pellegrini F, Di BP, Miselli V, et al. Impact of the "Diabetes Interactive Diary" telemedicine system on metabolic control, risk of hypoglycemia, and quality of life: a randomized clinical trial in type 1 diabetes. Diabetes Technol Ther 2013;15(8):670-9. PMID: 23844569.
23. Charpentier G, Benhamou PY, Dardari D, Clergeot A, Franc S, Schaepelynck-Belicar P, et al. The Diabeo software enabling individualized insulin dose adjustments combined with telemedicine support improves HbA1c in poorly controlled type 1 diabetic patients: a 6-month, randomized, open-label, parallel-group, multicenter trial (TeleDiab 1 Study). Diabetes Care 2011;34(3):533-9. PMID: 21266648.
24. Faridi Z, Liberti L, Shuval K, Northrup V, Ali A, Katz David L. Evaluating the impact of mobile telephone technology on type 2 diabetic patients' self-management: the NICHE pilot study. J Eval Clin Pract 2008;14(3):465-9. PMID: 18373577.

25. Nagrebetsky A, Larsen M, Craven A, Turner J, Mcrobert N, Murray E, et al. Stepwise self-titration of oral glucose-lowering medication using a mobile telephone-based telehealth platform in type 2 diabetes: a feasibility trial in primary care. J Diabetes Sci Technol 2013;7(1):123-34. PMID: 23439168.
26. Yoo HJ, Park MS, Kim TN, Yang SJ, Cho GJ, Hwang TG, et al. A ubiquitous chronic disease care system using cellular phones and the internet. Diabet Med 2009;26(6):628–35. PMID: 19538239.
