# Supplementary material for: The Efficacy of Mobile Phone Apps for Lifestyle Modification in Diabetes: Systematic Review and Meta-Analysis
Source: JMIR Mhealth Uhealth. 2019 Jan 15;7(1):e12297. doi: 10.2196/12297 (PMC6350094; doi:10.2196/12297)
Supplement: Multimedia Appendix 3 [file mhealth_v7i1e12297_app3.pdf]

Multimedia Appendix 3. Summary of HbA_{1c} data by study, by treatment arm, and by time

First author, year	Mean±SD of HbA_{1c} (%) for Intervention (upper) vs. Control (lower) at baseline and follow-up (months)							
	Baseline	3	4	5	6	9	10	12
Quinn et al (2008) [1]	9.51±NR	7.48±1.10						
	9.05±NR	8.37±1.33						
Quinn et al (2011) [2, 3]	9.9±2.1	7.8±1.3			7.5±1.2	7.7±2.1		7.9±1.7
	9.2±1.7	8.2±1.2			8.6±2.0	8.2±1.3		8.5±1.8
Kirwan et al (2013) [4]	9.08±1.18	8.32±0.84			7.97±0.73	7.80±0.75		
	8.47±0.86	8.23±0.89			8.43±1.00	8.58±1.16		
Orsama et al (2013) [5]	6.86±1.56						-0.40±0.61	
	7.09±1.51						0.036±0.63	
Holmen et al (2014) [6-8]	8.2±1.1		7.8±1.23					8.0±1.44
	8.3±1.2		8.0±1.23					8.2±1.33
Waki et al (2014) [9]	7.1±1.0	6.7±0.7						
	7.0±0.9	7.1±1.1						
Block et al (2015) [10]	5.6±0.3				-0.26±0.10			
	5.6±0.3				-0.18±0.10			
Fukuoka et al (2015) [11]	5.83±0.31			5.73±0.36				
	5.70±0.27			5.66±0.29				
Karhula et al (2015) [12]	7.25±NR							0.04±0.83

	7.20±NR			0.18±0.74
Skrøvseth et al (2015) [13]	8.33±0.87	-		
	8.06±1.32	0.63±0.58		
		-		
		0.57±0.78		
Wayne et al (2015) [14]	8.69±1.32	7.74±1.06	7.88±1.17	
	8.89±1.30	8.26±1.16	8.13±1.27	
Zhou et al (2016) [15]	9.86±2.38	7.91±1.58		
	9.76±2.51	8.97±2.08		
Bao et al (2017) [16]		8.23±1.89		
		9.39±2.59		
Rossi et al (2010) [17]	8.2±0.8	-0.5±0.8	-0.4±0.9	
	8.4±0.7	-0.4±0.6	-0.5±1.0	
Rossi et al (2013) [18]	8.4±0.79		7.9±0.79	
	8.5±0.80		8.1±0.80	
Charpentier et al (2011) [19]	9.19±1.14		8.63±1.07	
	8.91±0.90		9.10±1.16	
Faridi et al (2008) [20]	6,4±0.6	-0.1±0.3		
	6.5±0.7	0.3±1.0		
Nagrebetsky et al (2013) [21]				6.90±0.70
				7.50±1.40
Yoo et al (2009) [22]	7.6±0.9	7.1±0.8		
	7.4±0.9	7.6±1.0		

1. Quinn CC, Clough SS, Minor JM, Lender D, Okafor MC, Gruber-Baldini AL. WellDoc mobile diabetes management randomized

controlled trial: change in clinical and behavioral outcomes and patient and physician satisfaction. Diabetes Technol Ther 2008;10(3):160-8. PMID: 18473689.
2. Quinn CC, Shardell MD, Terrin MD, Barr EA, Ballew SH, Gruber-Baldini AL. Cluster-randomized trial of a mobile phone personalized behavioral intervention for blood glucose control. Diabetes care 2011;34(9):1934-42. PMID: 21788632.
3. Quinn CC, Sareh PL, Shardell ML, Terrin ML, Barr EA, Gruber-Baldini AL. Mobile diabetes intervention for glycemic control: impact on physician prescribing. J Diabetes Sci Technol 2014;8(2):362-70. PMID: 24876589.
4. Kirwan M, Vandelanotte C, Fenning A, Duncan MJ. Diabetes self-management smartphone application for adults with type 1 diabetes: randomized controlled trial. J Med Internet Res 2013;15(11):e235. PMID: 24225149.
5. Orsama AL, Lähteenmäki J, Harno K, Kulju M, Wintergerst E, Schachner H, et al. Active assistance technology reduces glycosylated hemoglobin and weight in individuals with type 2 diabetes: results of a theory-based randomized trial. Diabetes Technol Ther 2013;15(8):662-9. PMID: 23844570.
6. Holmen H, Torbjornsen A, Wahl AK, Jenum AK, Smastuen MC, Arsand E, et al. A mobile health intervention for self-management and lifestyle change for persons with type 2 diabetes, part 2: one-year results from the norwegian randomized controlled trial RENEWING HEALTH. JMIR Mhealth Uhealth 2014;2(4):e57. PMID: 25499872.
7. Torbjørnsen A, Jenum AK, Småstuen MC, Arsand E, Holmen H, Wahl AK, et al. A low-intensity mobile health intervention with and without health counseling for persons with type 2 diabetes, part 1: baseline and short-term results from a randomized controlled trial in the Norwegian part of RENEWING HEALTH. JMIR Mhealth Uhealth 2014;2(4):e52. PMID: 25499592.
8. Holmen H, Wahl A, Torbjørnsen A, Jenum AK, Småstuen MC, Ribu L. Stages of change for physical activity and dietary habits in persons with type 2 diabetes included in a mobile health intervention: the Norwegian study in RENEWING HEALTH. BMJ Open Diabetes Res Care 2016;4(1):e000193. PMID: 27239317.
9. Waki K, Fujita H, Uchimura Y, Omae K, Aramaki E, Kato S, et al. DialBetics: a novel smartphone-based self-management support system for type 2 diabetes patients. J Diabetes Sci Technol 2014;8(2):209-15. PMID: 24876569.
10. Block G, Azar K, Romanelli R, Block T, Hopkins D, Carpenter H, et al. Diabetes prevention and weight loss with a fully automated behavioral intervention by email, web, and mobile phone: a randomized controlled trial among persons with prediabetes. J Med Internet Res 2015;17(10):e240. PMID: 26499966.
11. Fukuoka Y, Gay C, Joiner K, Vittinghoff E. A novel diabetes prevention intervention using a mobile app: a randomized controlled trial

with overweight adults at risk. Am J Prev Med 2015;49(2):223-37. PMID: 26033349.
12. Karhula T, Vuorinen AL, Rääpysjärvi K, Pakanen M, Itkonen P, Tepponen M, et al. Telemonitoring and mobile phone-based health coaching among finnish diabetic and heart disease patients: randomized controlled trial. J Med Internet Res 2015;17(6):e153. PMID: 26084979.
13. Skrøvseth S, Årsand E, Godtliebsen F, Joakimsen R. Data-driven personalized feedback to patients with type 1 diabetes: a randomized trial. Diabetes Technol Ther 2015;17(7):482-9. PMID: 25751133.
14. Wayne N, Perez D, Kaplan D, Ritvo P. Health coaching reduces HbA1c in type 2 diabetic patients from a lower-socioeconomic status community: a randomized controlled trial. J Med Internet Res 2015;17(10):e224. PMID: 26441467.
15. Zhou W, Chen M, Yuan J, Sun Y. Welltang – a smart phone-based diabetes management application – improves blood glucose control in Chinese people with diabetes. Diabetes Res Clin Pract 2016;116:105-10. PMID: 27321324.
16. Bao S, Jiang H, Luo Y, Zhang D. Application of diabetes phone recipe software in diet intervention for patients with type 2 diabetes. Chinese Nursing Research 2017;31(11):1407-8. Doi: 10.3969/j.issn.1009-6493.2017.11.042.
17. Rossi MCE, Nicolucci A, Bartolo PD, Bruttomesso D, Girelli A, Ampudia FJ, et al. Diabetes interactive diary: a new telemedicine system enabling flexible diet and insulin therapy while improving quality of life: an open-label, international, multicenter, randomized study. Diabetes Care 2010;33(1):109-15. PMID: 19808926.
18. Rossi MCE, Nicolucci A, Lucisano G, Pellegrini F, Di BP, Miselli V, et al. Impact of the "Diabetes Interactive Diary" telemedicine system on metabolic control, risk of hypoglycemia, and quality of life: a randomized clinical trial in type 1 diabetes. Diabetes Technol Ther 2013;15(8):670-9. PMID: 23844569.
19. Charpentier G, Benhamou PY, Dardari D, Clergeot A, Franc S, Schaepelynck-Belicar P, et al. The Diabeo software enabling individualized insulin dose adjustments combined with telemedicine support improves HbA1c in poorly controlled type 1 diabetic patients: a 6-month, randomized, open-label, parallel-group, multicenter trial (TeleDiab 1 Study). Diabetes Care 2011;34(3):533-9. PMID: 21266648.
20. Faridi Z, Liberti L, Shuval K, Northrup V, Ali A, Katz David L. Evaluating the impact of mobile telephone technology on type 2 diabetic patients' self-management: the NICHE pilot study. J Eval Clin Pract 2008;14(3):465-9. PMID: 18373577.
21. Nagrebetsky A, Larsen M, Craven A, Turner J, Mcrobert N, Murray E, et al. Stepwise self-titration of oral glucose-lowering medication using a mobile telephone-based telehealth platform in type 2 diabetes: a feasibility trial in primary care. J Diabetes Sci Technol

2013;7(1):123-34. PMID: 23439168.
22. Yoo HJ, Park MS, Kim TN, Yang SJ, Cho GJ, Hwang TG, et al. A ubiquitous chronic disease care system using cellular phones and the internet. Diabet Med 2009;26(6):628–35. PMID: 19538239.
