# Supplementary material for: The Efficacy of Mobile Phone Apps for Lifestyle Modification in Diabetes: Systematic Review and Meta-Analysis
Source: JMIR Mhealth Uhealth. 2019 Jan 15;7(1):e12297. doi: 10.2196/12297 (PMC6350094; doi:10.2196/12297)
Supplement: Multimedia Appendix 4 [file mhealth_v7i1e12297_app4.pdf]

Multimedia Appendix 4. Overview of key functions of the Apps included

T1D

First author (year)	Physical activity monitoring	Dietary monitoring	Monitoring personnel	Health information and education	Medication adjustment support	Insulin bolus calculator	Clinical measurements logging	Feedback[a]	Freq. of HCP feedback	Type of HCP feedback
Kirwan et al (2013) [1]	Physical activities (minutes)	Food item in grams	Patient, Certified Diabetes Educator	√	√	×	BG	√(1,3)	Weekly	Text message
Skrøvseth et al (2015) [2]	Tailored step counter (for physical activity registration)	Eating habit registration, Photos of food and drink intake	Users, Healthcare givers	√	Not specified	√	BG	√(1)	N/A	N/A
Rossi et al (2010) [3]	Physical activity	Food intake	Patients, Physician	√	√	√	BG	√(2,3)	N/A	Text messages
Rossi	Physical	Food	Patients,	√	√	√	BG	√(2,3)	Every 1-	Text

First author (year)	Physical activity monitoring	Dietary monitoring	Monitoring personnel	Health information and education	Medication adjustment support	Insulin bolus calculator	Clinical measurements logging	Feedback[a]	Freq. of HCP feedback	Type of HCP feedback
et al (2013) [4]	activity	intake(CHO and calories)	Physician						3 weeks	messages
Charpentier et al (2011) [5]	Physical activity	Carbohydrate intake	Patients, investigators	×	√	√	BG	√(2,3)	Every two weeks	Telephone call

T2D

First author (year)	Physical activity monitoring	Dietary monitoring	Monitoring personnel	Health information and education	Medication adjustment support	Insulin bolus calculator	Clinical measurements logging	Feedback[a]	Freq. of HCP feedback	Type of HCP feedback
Quinn et al (2008) [6]	N/A	Carbohydrates intake	Research team, Patient, Physician	√	√	×	BG	√(2,3)	N/A	E-mail
Quinn et al	N/A	Carbohydrates intake	Patient, Healthcare	√	√	×	BG	√(2,3)	From every	Electronic

First author (year)	Physical activity monitoring	Dietary monitoring	Monitoring personnel	Health information and education	Medication adjustment support	Insulin bolus calculator	Clinical measurements logging	Feedback[a]	Freq. of HCP feedback	Type of HCP feedback
(2011) [7, 8]			provider						two months to one week	message, Telephone calls
Orsama et al (2013) [9]	Physical activity (pedometer)	N/A	Patients, Study nurse	√	Not specified	×	BP, body weight, BG (six patients with high HbA1c level)	√(1,2,3)	When necessary	N/A
Holmen et al (2014) [10-12]	Physical activity	Food intake	Patients	√	×	×	BG	√(1,2,3)	Each month for 4 months	Phone-based converations
Waki et al (2014) [13]	Pedometer counts, Type of exercise	Voice/text message about meals	Patients, Physician, Dietician	×	Not specified	×	BG, BP, body weight	√(1,2,3)	When necessary	N/A

First author (year)	Physical activity monitoring	Dietary monitoring	Monitoring personnel	Health information and education	Medication adjustment support	Insulin bolus calculator	Clinical measurements logging	Feedback[a]	Freq. of HCP feedback	Type of HCP feedback
	and its duration (for exercise isn't counted by a pedometer)	(main dish of the meal), Photos of meals								
Karhula et al (2015) [14]	Walking steps	N/A	Health coaches, Patients	√	×	×	BG, BP, Body weight	√(1,2,3)	Every 4 to 6 weeks	Phone-based conversation
Wayne et al (2015) [15]	Exercise frequency/ duration/intensity	Food intake (via photo journaling)	Patients, Health coach	×	Not specified	×	BG, Mood	√(3)	Any time in the 24-hour cycle	Via secure messaging, scheduled phone contact

First author (year)	Physical activity monitoring	Dietary monitoring	Monitoring personnel	Health information and education	Medication adjustment support	Insulin bolus calculator	Clinical measurements logging	Feedback[a]	Freq. of HCP feedback	Type of HCP feedback and/or during in-person meetings
Weegen et al (2015) [16]	Three-dimensional (3D) activity monitor	N/A	Patients, Practice nurse	√	×	Not specified	N/A	√(1,2,3)	First week, after 2 weeks, after 2-3 months and after 4-6 months	Off-line individual consultations
Plotnikoff et al (2017) [17]	Self-monitoring the progress of weekly	N/A	Patients	√	×	×	N/A	N/A	N/A	N/A

First author (year)	Physical activity monitoring	Dietary monitoring	Monitoring personnel	Health information and education	Medication adjustment support	Insulin bolus calculator	Clinical measurements logging	Feedback[a]	Freq. of HCP feedback	Type of HCP feedback
	physical activity goals									
Bao et al (2017) [18]	Physical activity	Dietary intake	Patients, Research team	√	×	×	BG	√(3)	One or two times a week	The outpatient follow-up, Phone calls
Faridi et al (2008) [19]	Physical activity (pedometer)	N/A	Patients, Healthcare providers	√	×	×	BG, Body weight	√(1,2)	N/A	N/A
Nagrebetsky et al (2013) [20]	N/A	N/A	Patients, Research nursing staff	×	√	×	BG	√(1,3)	Monthly	Telephone calls
Yoo et	Exercise	N/A	Patients,	√	Not	×	BG, BP,	√(1,2,3)	When	Send

First author (year)	Physical activity monitoring	Dietary monitoring	Monitoring personnel	Health information and education	Medication adjustment support	Insulin bolus calculator	Clinical measurements logging	Feedback[a]	Freq. of HCP feedback	Type of HCP feedback
al (2009) [21]	time		Physicians		specified		Body weight		necessary	text messages through the system

GDM

First author (year)	Physical activity monitoring	Dietary monitoring	Monitoring personnel	Health information and education	Medication adjustment support	Insulin bolus calculator	Clinical measurements logging	Feedback[a]	Freq. of HCP feedback	Type of HCP feedback
Kennelly et al (2018) [22]	N/A	N/A	N/A	√	×	×	N/A	√(3)	Every 2 weeks (emails), 28 and 34 weeks of gestation	Emails, Face-to-face hospital visits

Prediabetes

First author (year)	Physical activity monitoring	Dietary monitoring	Monitoring personnel	Health information and education	Medication adjustment support	Insulin bolus calculator	Clinical measurements logging	Feedback[a]	Freq. of HCP feedback	Type of HCP feedback
Block et al (2015) [23]	Activity	Dietary intake	Patients	√	×	×	Body weight	√(1,2)	N/A	N/A
Fukuoka et al (2015) [24]	Total number of steps per day, The types and duration of physical activities	Total daily caloric intake	Patients	√	×	×	Body weight	√(2)	N/A	N/A
Spring et al (2017) [25]	MVPA (moderate-to-vigorous intensity physical activity)	Dietary intake	Patients, Coaches	√	×	×	Body weight	√(1,3)	Weekly for 6 months (messages). Weekly	Personalized messages, Phone calls

First author (year)	Physical activity monitoring	Dietary monitoring	Monitoring personnel	Health information and education	Medication adjustment support	Insulin bolus calculator	Clinical measurements logging	Feedback[a]	Freq. of HCP feedback	Type of HCP feedback
									for first eight weeks and monthly from months 3-6 (phone calls)	

T1D and T2D

First author (year)	Physical activity monitoring	Dietary monitoring	Monitoring personnel	Health information and education	Medication adjustment support	Insulin bolus calculator	Clinical measurements logging	Feedback[a]	Freq. of HCP feedback	Type of HCP feedback
Zhou et al (2016) [26]	N/A	Carbohydrate intake	Patients, Research team	√	√	Not specified	BG, BP	√(1,2,3)	Once a week or every two	N/A

First author (year)	Physical activity monitoring	Dietary monitoring	Monitoring personnel	Health information and education	Medication adjustment support	Insulin bolus calculator	Clinical measure ments logging	Feedback[a]	Freq. of HCP feedback weeks	Type of HCP feedback

[a]1: Graphical feedback; 2. Automated feedback; 3. HCP feedback

1. Kirwan M, Vandelanotte C, Fenning A, Duncan MJ. Diabetes self-management smartphone application for adults with type 1 diabetes: randomized controlled trial. J Med Internet Res 2013;15(11):e235. PMID: 24225149.
2. Skrøvseth S, Årsand E, Godtliebsen F, Joakimsen R. Data-driven personalized feedback to patients with type 1 diabetes: a randomized trial. Diabetes Technol Ther 2015;17(7):482-9. PMID: 25751133.
3. Rossi MCE, Nicolucci A, Bartolo PD, Bruttomesso D, Girelli A, Ampudia FJ, et al. Diabetes interactive diary: a new telemedicine system enabling flexible diet and insulin therapy while improving quality of life: an open-label, international, multicenter, randomized study. Diabetes Care 2010;33(1):109-15. PMID: 19808926.
4. Rossi MCE, Nicolucci A, Lucisano G, Pellegrini F, Di BP, Miselli V, et al. Impact of the "Diabetes Interactive Diary" telemedicine system on metabolic control, risk of hypoglycemia, and quality of life: a randomized clinical trial in type 1 diabetes. Diabetes Technol Ther 2013;15(8):670-9. PMID: 23844569.
5. Charpentier G, Benhamou PY, Dardari D, Clergeot A, Franc S, Schaepelynck-Belicar P, et al. The Diabeo software enabling individualized insulin dose adjustments combined with telemedicine support improves HbA1c in poorly controlled type 1 diabetic patients: a 6-month, randomized, open-label, parallel-group, multicenter trial (TeleDiab 1 Study). Diabetes Care 2011;34(3):533-9. PMID: 21266648.
6. Quinn CC, Clough SS, Minor JM, Lender D, Okafor MC, Gruber-Baldini AL. WellDoc mobile diabetes management randomized controlled trial: change in clinical and behavioral outcomes and patient and physician satisfaction. Diabetes Technol Ther 2008;10(3):160-8. PMID: 18473689.

7. Quinn CC, Shardell MD, Terrin MD, Barr EA, Ballew SH, Gruber-Baldini AL. Cluster-randomized trial of a mobile phone personalized behavioral intervention for blood glucose control. Diabetes Care 2011;34(9):1934-42. PMID: 21788632.
8. Quinn CC, Sareh PL, Shardell ML, Terrin ML, Barr EA, Gruber-Baldini AL. Mobile diabetes intervention for glycemic control: impact on physician prescribing. J Diabetes Sci Technol 2014;8(2):362-70. PMID: 24876589.
9. Orsama AL, Lähteenmäki J, Harno K, Kulju M, Wintergerst E, Schachner H, et al. Active assistance technology reduces glycosylated hemoglobin and weight in individuals with type 2 diabetes: results of a theory-based randomized trial. Diabetes Technol Ther 2013;15(8):662-9. PMID: 23844570.
10. Holmen H, Torbjornsen A, Wahl AK, Jenum AK, Smastuen MC, Arsand E, et al. A mobile health intervention for self-management and lifestyle change for persons with type 2 diabetes, part 2: one-year results from the norwegian randomized controlled trial RENEWING HEALTH. JMIR Mhealth Uhealth 2014;2(4):e57. PMID: 25499872.
11. Torbjørnsen A, Jenum AK, Småstuen MC, Arsand E, Holmen H, Wahl AK, et al. A low-intensity mobile health intervention with and without health counseling for persons with type 2 diabetes, part 1: baseline and short-term results from a randomized controlled trial in the Norwegian part of RENEWING HEALTH. JMIR Mhealth Uhealth 2014;2(4):e52. PMID: 25499592.
12. Holmen H, Wahl A, Torbjørnsen A, Jenum AK, Småstuen MC, Ribu L. Stages of change for physical activity and dietary habits in persons with type 2 diabetes included in a mobile health intervention: the Norwegian study in RENEWING HEALTH. BMJ Open Diabetes Res Care 2016;4(1):e000193. PMID: 27239317.
13. Waki K, Fujita H, Uchimura Y, Omae K, Aramaki E, Kato S, et al. DialBetics: a novel smartphone-based self-management support system for type 2 diabetes patients. J Diabetes Sci Technol 2014;8(2):209-15. PMID: 24876569.
14. Karhula T, Vuorinen AL, Rääpysjärvi K, Pakanen M, Itkonen P, Tepponen M, et al. Telemonitoring and mobile phone-based health coaching among finnish diabetic and heart disease patients: randomized controlled trial. J Med Internet Res 2015;17(6):e153. PMID: 26084979.
15. Wayne N, Perez D, Kaplan D, Ritvo P. Health coaching reduces HbA1c in type 2 diabetic patients from a lower-socioeconomic status community: a randomized controlled trial. J Med Internet Res 2015;17(10):e224. PMID: 26441467.
16. Weegen S, Verwey R, Spreeuwenberg M, Tange H, Weijden T, Witte L. It's LiFe! Mobile and web-based monitoring and feedback tool embedded in primary care increases physical activity: a cluster randomized controlled trial. J Med Internet Res 2015;17(7):e184. PMID: 26209025.

17. Plotnikoff RC, Wilczynska M, Cohen KE, Smith JJ, Lubans DR. Integrating smartphone technology, social support and the outdoor physical environment to improve fitness among adults at risk of, or diagnosed with, type 2 diabetes: findings from the 'eCoFit' randomized controlled trial. Prev Med 2017;105:404-11. PMID: 28887192.
18. Bao S, Jiang H, Luo Y, Zhang D. Application of diabetes phone recipe software in diet intervention for patients with type 2 diabetes. Chinese Nursing Research 2017;31(11):1407-8. Doi: 10.3969/j.issn.1009-6493.2017.11.042.
19. Faridi Z, Liberti L, Shuval K, Northrup V, Ali A, Katz David L. Evaluating the impact of mobile telephone technology on type 2 diabetic patients' self-management: the NICHE pilot study. J Eval Clin Pract 2008;14(3):465-9. PMID: 18373577.
20. Nagrebetsky A, Larsen M, Craven A, Turner J, Mcrobert N, Murray E, et al. Stepwise self-titration of oral glucose-lowering medication using a mobile telephone-based telehealth platform in type 2 diabetes: a feasibility trial in primary care. J Diabetes Sci Technol 2013;7(1):123-34. PMID: 23439168.
21. Yoo HJ, Park MS, Kim TN, Yang SJ, Cho GJ, Hwang TG, et al. A ubiquitous chronic disease care system using cellular phones and the internet. Diabet Med 2009;26(6):628–35. PMID: 19538239.
22. Kennelly MA, Ainscough K, Lindsay KL, O'Sullivan E, Gibney ER, McCarthy M, et al. Pregnancy exercise and nutrition with smartphone application support: a randomized controlled trial. Obstet Gynecol 2018;131(5):818-26. PMID: 29630009.
23. Block G, Azar K, Romanelli R, Block T, Hopkins D, Carpenter H, et al. Diabetes prevention and weight loss with a fully automated behavioral intervention by email, web, and mobile phone: a randomized controlled trial among persons with prediabetes. J Med Internet Res 2015;17(10):e240. PMID: 26499966.
24. Fukuoka Y, Gay C, Joiner K, Vittinghoff E. A novel diabetes prevention intervention using a mobile app: a randomized controlled trial with overweight adults at risk. Am J Prev Med 2015;49(2):223-37. PMID: 26033349.
25. Spring B, Pellegrini CA, Pfammatter A, Duncan JM, Pictor A, McFadden HG, et al. Effects of an abbreviated obesity intervention supported by mobile technology: the ENGAGED randomized clinical trial. Obesity 2017;25(7):1191-8. PMID: 28494136.
26. Zhou W, Chen M, Yuan J, Sun Y. Welltang – a smart phone-based diabetes management application – improves blood glucose control in Chinese people with diabetes. Diabetes Res Clin Pract 2016;116:105-10. PMID: 27321324.
